# Virological Blips and Predictors of Post Treatment Viral Control After Stopping ART Started in Primary HIV Infection

**DOI:** 10.1097/QAI.0000000000001220

**Published:** 2016-11-08

**Authors:** Sarah Fidler, Ashley D. Olson, Heiner C. Bucher, Julie Fox, John Thornhill, Charles Morrison, Roberto Muga, Andrew Phillips, John Frater, Kholoud Porter

**Affiliations:** * Department of Genitourinary Medicine and Infectious Disease, Imperial College, London, United Kingdom;; † University College London, London, United Kingdom;; ‡ Medical Research Council Clinical Trials Unit at University College London, London, UK. Basel Institute for Clinical Epidemiology and Biostatistics, University Hospital Basel, Basel, Switzerland;; § Guys and St Thomas Hospital NHS Trust, London, United Kingdom;; ‖ Clinical and Epidemiological Sciences, FHI 360, Durham, NC;; ¶ Department of Internal Medicine, Hospital Universitari Germans Trias i Pujol, Badalona, Spain; and; # Peter Medawar Building for Pathogen Research, Nuffield Department of Medicine, Oxford University, United Kingdom; Oxford Martin School, Oxford, United Kingdom; Oxford NIHR Biomedical Research Centre, Oxford, United Kingdom.

**Keywords:** cure, viral blips, primary HIV infection, post treatment control (PTC)

## Abstract

**Background:**

Few individuals commencing antiretroviral therapy (ART) in primary HIV infection (PHI) maintain undetectable viremia after treatment cessation. Associated factors remain unclear given the importance of the phenomenon to cure research.

**Methods:**

Using CASCADE data of seroconverters starting ART in PHI (≤6 months from seroconversion), we estimated proportions experiencing viral blips (>400 copies followed by <400 copies HIV-RNA/mL without alteration of regimen) while on ART. We used Cox models to examine the association between time from ART stop to loss of control (2 consecutive measurements >1000 copies per milliliter) and magnitude and frequency of blips while on ART, time from seroconversion to ART, time on ART, adjusting for mean number of HIV-RNA measurements/year while on ART, and other confounders.

**Results:**

Seven hundred seventy-eight seroconverters started ART in PHI with ≥3 HIV-RNA measurements. Median interquartile range (IQR) ART duration was 16.2 (8.0–35.9) months, within which we observed 13% with ≥1 blip. Of 228 who stopped ART, 119 rebounded; time to loss of control was associated with longer interval between seroconversion and ART initiation [hazard ratio (HR) = 1.16 per month; 1.04, 1.28], and blips while on ART (HR = 1.71 per blip; 95% confidence interval = 0.94 to 3.10). Longer time on ART (HR = 0.84 per additional month; 0.76, 0.92) was associated with lower risk of losing control. Of 228 stopping ART, 22 (10%) maintained post treatment control (PTC), ie, HIV-RNA <50 copies per milliliter ≥24 months after ART cessation.

**Conclusion:**

HIV viral blips on therapy are associated with subsequent viral rebound on stopping ART among individuals treated in PHI. Longer duration on ART is associated with a greater chance of PTC.

## INTRODUCTION

Effective combination antiretroviral therapy (ART) controls HIV-1 viral replication to levels below the limit of detection of current laboratory assays,^1–3^ confers improved clinical outcome,^4^ and prevents onward transmission.^5^ However, during suppressive therapy many patients experience transient detectable viremia, or “blips,”^6^ defined as detectable plasma viremia >50 copies HIV-RNA/mL which subsequently returns to <50 copies without alteration of ART regimen.^7,8^ Among such individuals subsequent viral failure remains infrequent if blip levels remain low^6,9^ but, where virological failure ensued, the best predictor was a blip magnitude of >400 copies HIV-1 RNA/mL.^10,11^ Furthermore, for most patients achieving HIV-RNA <50 copies per milliliter, approximately 1–3 copies of plasma HIV-RNA can be detected using more sensitive assays.^12^

ART is not a cure for HIV-1 infection—a consequence of an inaccessible reservoir of virally infected cells.^13–15^ Novel approaches exploring “HIV-cure” strategies are under development. At present, although not routinely recommended, the only true test of “cure or remission” within the context of these trials is to stop ART, but only where planned and carefully monitored. It remains uncertain which individuals might be best placed to safely interrupt therapy.

For rare individuals initiating ART in primary HIV infection (PHI), plasma viremia remains undetectable after treatment interruption (TI). This phenotype has been termed post treatment control (PTC)^16^ and seems to be more common among individuals stopping treatment initiated during PHI; a disease stage where the viral reservoir is smaller compared with chronic infection,^17,18^ where immune dysfunction is less^19^ and ART induced immunological recovery is often better.^20^ Assessing PTC necessarily requires a TI. For most individuals, a TI results in viral load rebound,^21–23^ which is more rapid among those initiating in chronic infection than in PHI. Furthermore, although this rebound has been shown to confer an increased risk of all-cause mortality for those interrupting ART initiated in chronic infection,^15^ viral recrudescence increases the risk of onward transmission after TI, irrespective of disease stage. Therefore, if TI is planned in the context of cure research, it needs to be HIV-RNA guided and closely monitored, as prolonged TI guided by CD4 has been shown to increase morbidity/mortality.^15^ Predictive markers that can evaluate individuals at increased likelihood of achieving PTC will be valuable tools in the design of future cure trials.

Although the exact mechanisms underlying PTC remain unknown, important predictors include low levels of viral reservoirs before TI, early initiation of ART, and longer duration of therapy.^16^ This is supported by data from the SPARTAC trial^24,25^ where pre-TI levels of HIV-1 DNA also predicted viral rebound^26^ after ART cessation and data from early treatment studies in primates.^27^

The source and mechanism for viral blips remains uncertain; however,^28^ and although blips may reflect transient periods of reduced ART adherence,^29,30^ or variations between viral load assays,^31^ the frequency and magnitude of blips on ART might also be related to the size of the proviral reservoir^32,33^ and intermittent immune activation.^34,35^ We, therefore, explored the frequency, magnitude, and predictive value of measured viral blips on the probability of achieving PTC among a cohort of treated HIV-1 seroconverters interrupting ART started initiated in PHI.

## METHODS

### Data Source

We used pooled data from the CASCADE 2014 data release in EuroCoord (www.EuroCoord.net) of seroconverter cohorts across Europe, Australia, Canada, and Sub-Saharan Africa. The collaboration has been previously described,^36^ in brief date of HIV seroconversion in CASCADE is estimated most commonly as the midpoint between the last documented HIV negative and the first HIV-positive antibody test dates with an interval of ≤3 years between the 2 dates (87%). Dates of seroconversion for the remaining individuals (10%) is estimated through laboratory evidence of acute infection (HIV DNA polymerase chain reaction positivity in the absence of HIV antibodies or antigen positivity with <4 bands on Western blot), or as the date of HIV seroconversion illness with both an earlier documented negative and a later positive HIV test not more than 3 years apart (2%). Fiebig staging is not part of the algorithm for estimating date of seroconversion.^37^

All cohorts contributing to CASCADE received ethics approval from their individual ethics review boards.

### Inclusion Criteria

Only adults older than 16 years starting ART within 6 months of estimated HIV seroconversion (PHI) with at least 3 HIV-RNA measurements while on ART were eligible for this analysis. Eligibility criteria and numbers, therefore, differ from our previous publication on proportions achieving PTC.^20^

### Blips

We characterized the proportion of individuals experiencing blips while on ART initiated in PHI, and the associated exact 95% confidence intervals (CIs) for binomial distributed data. We also identified individuals with multiple blips while on ART. We used a modified definition of blip as a single plasma HIV-RNA measure >400 copies per milliliter in a previously suppressed individual followed by subsequent viral suppression (<400 copies per milliliter) without change in ART regimen.^1^ Any magnitude of viremia episode was considered as a blip, as we were interested in the effect of blips regardless of the reasons for them. To be classified as having a blip or not, we included only individuals with HIV-RNA measured with assays detecting ≤400 copies per milliliter. Periods of unsuppressed viremia occurring during ART changes were attributed to the change in regimen and did not contribute to the analysis of blip rates.

In a sensitivity analysis on blip definitions, we defined additional blip thresholds of HIV-RNA >50, >100, and >200 copies per milliliter. The number of individuals included in this sensitivity analysis was smaller than the numbers included in the main analysis as fewer individuals were measured with assays detecting lower values.

### Loss of Viremic Control

We used Kaplan–Meier methods to describe time from ART cessation to loss of viremic control and examined associated factors using Cox proportional hazards models. Loss of control was defined as the second of 2 consecutive HIV-1 RNA measurements >1000 copies per milliliter. Factors of interest were time on ART, time between HIV-1 seroconversion to ART initiation, plasma HIV-RNA at seroconversion, ART initiation year, CD4 T-cell count at ART initiation, CD4 T-cell count at ART cessation, ART class, age at HIV-1 seroconversion, sex, HIV-1 transmission risk group, and magnitude and frequency of blips while on ART. As rebound is more likely to be observed in those with more frequent measurements, we also adjusted for the mean number of HIV-RNA measurements/year while on ART. This also served as a proxy for adherence and engagement in care. Linear terms for all continuous variables were used, as there was no evidence for departures from linearity using natural cubic splines.^38^

We preformed several sensitivity analyses for the analysis of loss of viremic control. We defined blips as >50, >100, and >200 copies per milliliter, and we included covariates on the magnitude and frequency of each blip threshold. We also defined loss of control as the second of 2 consecutive HIV-RNA measurements greater than the given blip threshold. In additional, we limited our analysis to individuals who were on ART for at least 1 year before stopping treatment.

### Post Treatment Controllers

PTC was defined as remaining <50 copies per milliliter for at least 24 months after ART stops. Once PTC was achieved, we used a strict definition for loss of PTC status as the first of 2 consecutive HIV-RNA measurements >50 copies per milliliter. Because there were very few PTCs, we did not formally analyze factors related to post treatment control.

## RESULTS

### Baseline Characteristics

Of 31,772 individuals in CASCADE, 22,688 were defined as PHI in the ART era (≥1995). Of these, 778 started ART within 6 months of seroconversion and had at least 3 HIV-RNA measurements. Of these, 228 (30%) subsequently stopped ART; reasons for stopping ART are unknown.

Among the 778 individuals starting ART in PHI, the majority were male (92%) seroconverting between 1995 and 2013 at median (IQR) age of 34 (28–42) years. Risk factors for HIV-1 infection were sex between men (75%), sex between men and women (17%), injecting drug use (4%), or other/unknown (5%). ART regimens included Nucleoside/Nucleotide Reverse Transcriptase Inhibitors NRTI backbone with protease inhibitor (PI) based (45%) or nonnucleoside reverse-transcriptase inhibitor based (37%) and other triple combinations (18%). Median interquartile range (IQR) time to ART initiation from seroconversion was 2.3 (0.7–4.1) months and median (IQR) time spent on ART initiated in PHI was 16.2 (8.0–35.9) months. Initial HIV-RNA measurement after HIV diagnosis was median 5.3 (4.5–5.9) log_10_ copies per milliliter and median CD4 at ART initiation was 477 (316–658) cells per cubic millimeter, Table 1.

**TABLE 1.**
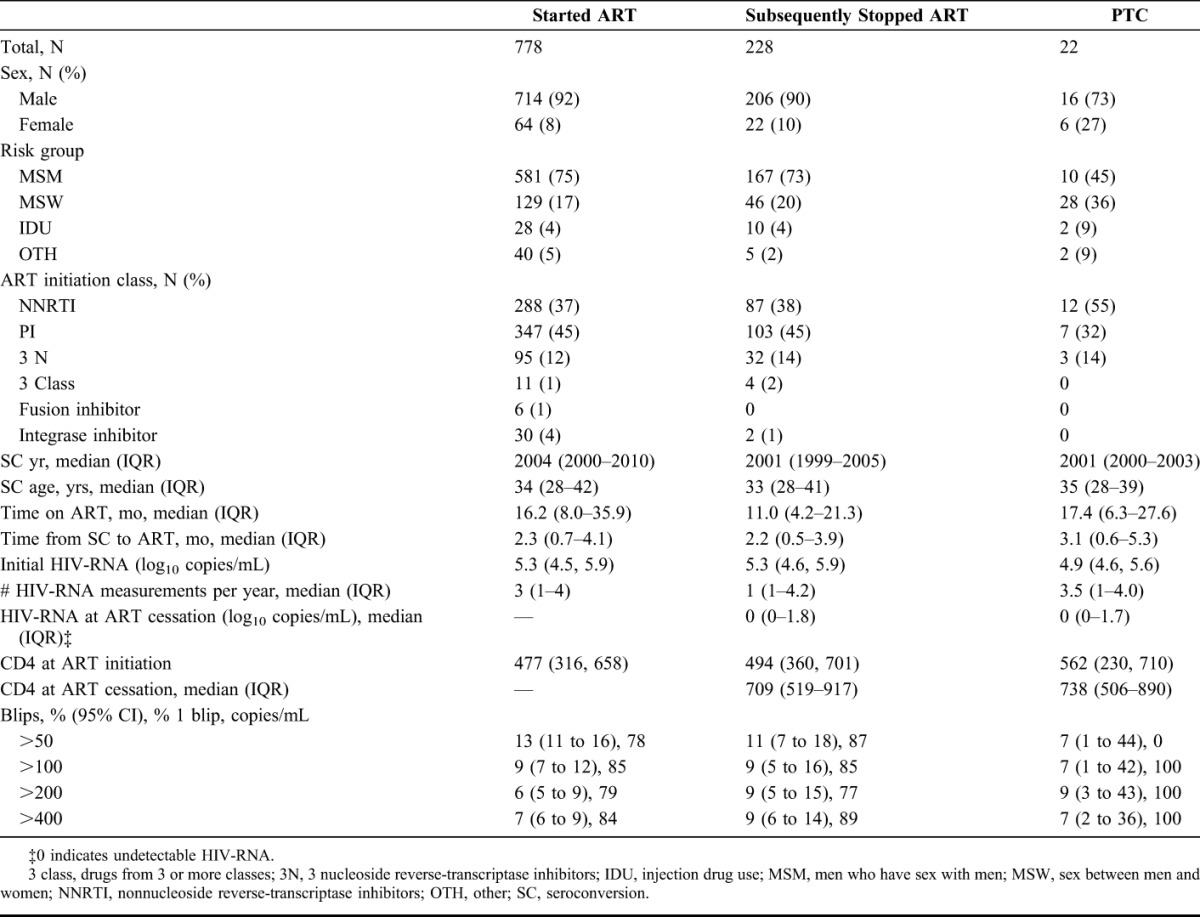
Baseline Characteristics of Individuals Initiating ART Within 6 Months of HIV-1 Seroconversion, Those Subsequently Stopping ART, and Post Treatment Controllers (PTC) in CASCADE

Baseline characteristics for the subset of individuals subsequently stopping ART initiated in PHI (n = 228) were similar to all those starting ART in PHI (n = 778), with the exception of seroconversion year and time spent on ART, as those subsequently stopping ART seroconverted in slightly earlier years, median (IQR) 2001 (1999–2005), and spent slightly less time on ART, median 11.0 (4.2–21.3) months. Blip rates were similar among individuals starting ART in PHI and individuals subsequently interrupting therapy, Table 1.

### Blips While on ART

Of those starting ART in PHI with HIV-1 plasma HIV-RNA measured using assays detecting ≤400 copies per milliliter, we observed 7% (95% CI: 6 to 9) of individuals with 1 blip over 400 copies per milliliter, the majority (84%) of whom we observed only 1 blip. Among those that blipped over 400 copies per milliliter, median (IQR) time to the first blip was 1.0 (0.6–2.5) year and, among those with multiple blips, median (IQR) time between blips was 0.7 (0.6–1.1) years. Median (IQR) time to recover from a blip was 57 (32–111) days. Similarly, we observed at least 1 blip in 13% (11–16), 9% (7, 12), and 6% (5, 9) over 50, 100, and 200 copies per milliliter, respectively, and the majority, again, of whom we observed only 1 blip. Blip rates were similar among those who subsequently stopped ART, Table 2.

**TABLE 2.**
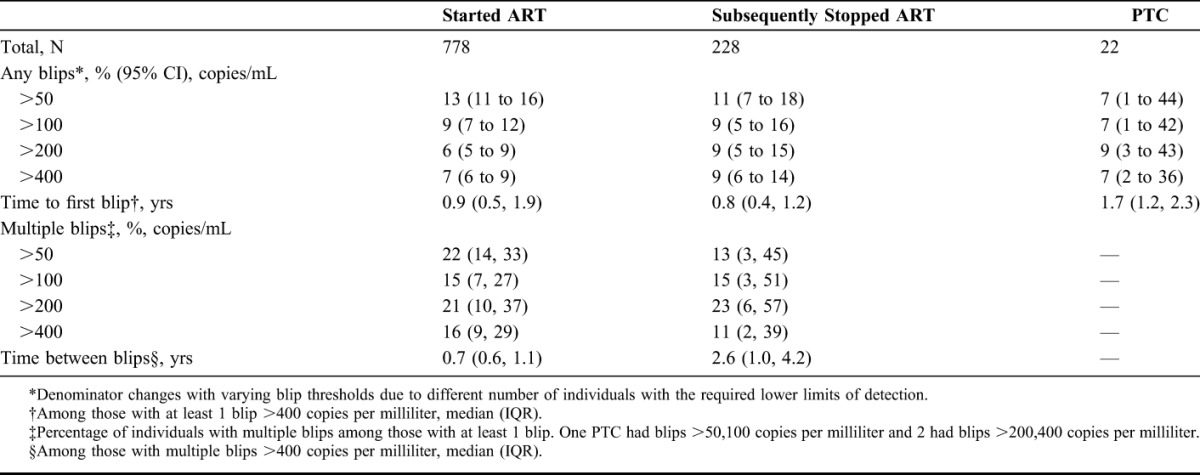
Characteristics of Blips Among Individuals Initiating ART Within 6 Months of HIV-1 Seroconversion, Those Subsequently Stopping ART, and Post Treatment Controllers (PTC) in CASCADE

### Factors Associated With Loss of Control After Stopping ART

Among the 228 individuals stopping ART, 22 (10%) individuals fulfilled the definition of PTC. Viral rebound was observed in 119 (52%) individuals; 23%, 37%, and 45% were observed to have rebounded by 3, 6, and 9 months, respectively. Median (95% CI) time to rebound was 10.3 (7.6 to 16.4) months. Several factors were independently associated with loss of control. Each blip >400 copies per milliliter was associated with a 71% increased risk of loss of control [hazard ratio (HR) = 1.71 (0.94, 3.10)], as was longer interval between seroconversion and ART initiation [HR = 1.16 per additional month (1.04, 1.28)]. More frequent HIV-RNA measurements while on ART were also associated with loss of control [HR = 1.10 per mean additional measurement/year increase (1.02, 1.17)] (Table 3).

**TABLE 3.**
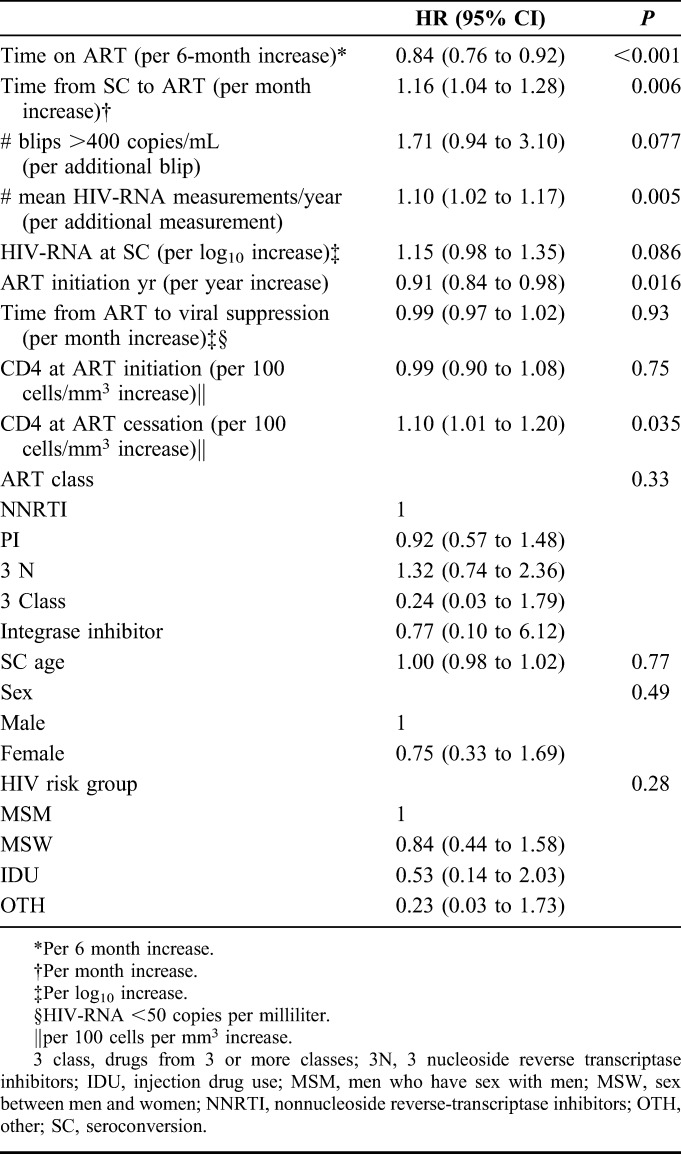
Multivariable Analysis of the Factors Associated With Virologic Rebound Among Those Stopping ART Initiated Within 6 Months of HIV Seroconversion Using the CASCADE Dataset

Conversely, longer time spent on ART was independently associated with a decreased risk in loss of control [HR (95% CI) = 0.84 per 6 month increase (0.76 to 0.92)], as was later year of ART initiation [HR = 0.91 (0.84, 0.98)] (Table 3). There was no evidence of an association between loss of control and CD4 T-cell count at ART initiation, ART initiation class, seroconversion age, sex, or HIV-1 transmission risk group.

**FIGURE 1.**
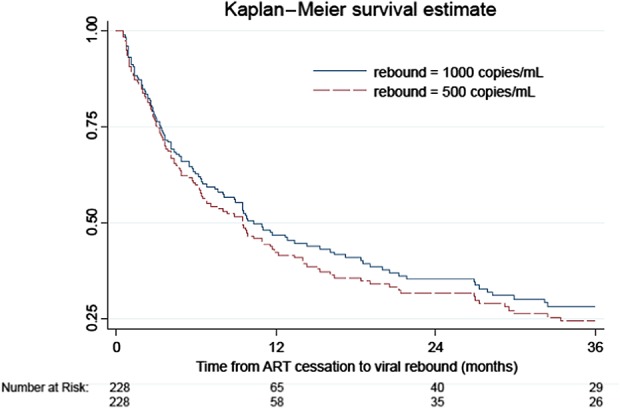
Time from ART cessation to virologic rebound, defined as HIV-RNA ≥500, 1000 copies per mL, among those stopping ART initiated within 6 months of HIV seroconversion in CASCADE.

Using different blip thresholds, we observed an increased risk of loss of virologic control per increase in number of blips of similar magnitude to the results presented for blips >400 copies per milliliter in Table 1, although this did not reach statistical significance as fewer individuals contributed to these analyses. For each additional blip we found, HR = 1.96 (0.71, 5.38), 1.66 (0.88, 3.13), and 1.65 (0.90, 3.05) for blips of >50, 100, and 200 copies per milliliter, respectively. Defining loss of control as HIV-RNA >500 copies per milliliter resulted in similar time to rebound (Fig. 1), and factors associated with rebound remained the same as for the main analysis (data not shown). Time from the start of ART to the first blip was not associated with time to virologic rebound (data not shown).

Restricting to individuals who had been on ART for a year or more before stopping reduced the number of individuals included in analysis to 91. Time spent on ART and number of blips >400 copies per milliliter retained the same magnitude of association, as in the main analysis, although no longer remained statistically significant effects for time spent on ART or number of blips >400 copies per milliliter [HR = 0.90 (0.78, 1.03) and 2.31 (0.71, 7.48), respectively].

### Post Treatment Controllers

Of the 228 individuals interrupting ART, 22 (10.3%) achieved PTC status. ART initiation combinations for these 22 PTCs included nonnucleoside reverse-transcriptase inhibitor-based (n = 12; 55%), PI-based (n = 7; 32%), or triple nucleoside reverse-transcriptase inhibitors (3N) (n = 3; 14%) regimens. The proportion of PTCs for who we observed blips while on ART was slightly lower compared with the 206 individuals interrupting ART not achieving PTC status. We observed only 1 PTC with a blip >50 copies per milliliter, compared with 14 [12% (7, 19)] of all other individuals. Post treatment controllers also spent slightly longer time on ART compared with all other individuals interrupting ART, median (IQR) 17.4 (6.3–27.6) months for PTCs compared with 10.9 (3.6–19.0) months. The first HIV-RNA measurement after HIV diagnosis was slightly lower among PTCs with a median (IQR) 4.9 (4.6–5.6) log_10_ copies per milliliter compared with 5.3 (4.6–5.9) log_10_ copies per milliliter and the CD4 at ART initiation was slightly higher among PTCs with a median (562, 230–710) cells per mm^3^ compared with 493 (363–690) cells per mm^3^ among the remaining 206 individuals interrupting ART. Median number of HIV-RNA measurements per year after ART interruption was similar among PTCs and non-PTCs at 1 measure/year. Heterogeneity in time from HIV-1 seroconversion to ART initiation was small because of the inclusion criteria of starting ART within 6 months of HIV seroconversion and was, therefore, similar between post treatment controllers and all other individuals.

## DISCUSSION

Using the large CASCADE dataset of individuals with well-estimated dates of HIV seroconversion, we provide the first evidence that frequency and magnitude of viral blips while on ART initiated in PHI is associated with viral rebound among individuals interrupting ART started in PHI.

The prevalence of PTC (defined by 2 years of undetectable viremia after TI) in our cohort is estimated to be 10.3%. This is not dissimilar to other cohorts reporting PTC^23,39–41^ and slightly lower than the VISCONTI study (15.6%).^16^ That said, most cohorts report few or none, including among early treated populations.^42–46^ In comparison with VISCONTI, the duration of ART was shorter in our cohort, but shorter time from HIV diagnosis to ART initiation was also predictive of PTC in both cohorts.

Although much data exist for the predictive value of blips on subsequent viral failure among individuals on ART in chronic stages of HIV disease,^47–49^ it is difficult to extrapolate this to PTC. The source of viral blips on ART is unclear. They may, for example, represent release of virus from transient, random activation of latently infected cells,^34^ fluctuations in levels of persistent viral replication on ART,^50^ sanctuary sites of suboptimal antiretroviral penetrance,^51^ or nonadherence to ART regimens. One explanation for our findings is that initiating ART early in PHI results in fewer viral blips of lower magnitude because of the smaller HIV-1 viral reservoir achieved among these individuals.^52,53^ Unfortunately, samples were not available to determine HIV-1 DNA measurements to test this assumption, although this is consistent with data from SPARTAC showing that levels of total HIV-1 DNA measured at TI predict time to loss of control.^24^

The associations observed in our cohort between timing of ART initiation, duration of therapy, and PTC were linear and, accordingly, we were unable to determine an optimal period beyond which ART initiation after seroconversion may be too late to achieve PTC. These are key questions that need to be addressed in prospective studies to inform future cure trial designs and help develop algorithms to predict likelihood of PTC. Of note, while HIV-1 HIV-RNA and initial CD4 count measurement at diagnosis of PHI are known to predict disease progression,^54^ for those initiating immediate ART in our cohort, these parameters did not appear to influence subsequent PTC status, suggesting that the mechanisms underpinning the 2 processes may be different.

Host genetic factors may also determine PTC status, although we were not able to explore these factors. In the VISCONTI cohort there was no evidence for enrichment of protective HLA Class I alleles and only weak HIV-specific immunity was observed.

The data presented from this large cohort should be interpreted within the limitations of any observational study. First, for those individuals with a measurable viral blip, we assumed that ART was continuous through this period and a viral blip is not the result of temporary poor adherence, absorption, or the assay used; however, irrespective of the cause, the presence of a blip predicted viral rebound and hence must be incorporated into any algorithm for future HIV cure trials.^55^ Second, reasons for ART initiation and subsequent cessation for eligible individuals are unknown and those stopping may differ in important characteristics from those not stopping, although short-course ART in PHI was not an uncommon treatment strategy by a number of clinicians during the time.^24,39,40^ In any case, baseline HIV-RNA and CD4 measurements at ART initiation were similar for those subsequently stopping and those not stopping ART. It is, therefore, unlikely that reasons for stopping ART initiated in PHI were related to outcome but we acknowledge, as with all observational studies, that unmeasured confounding factors may remain, including in the choice of whether or not to initiate ART in PHI. Third, the absence of data on ART adherence is a limitation of these analyses, and blips may, therefore be, as a result of periods of nonadherence or viral breakthrough. We included the number viral load measurements as a surrogate of adherence in the multivariate analyses. In any case, our findings are of clinical relevance to clinicians as they highlight that patients experiencing blips, regardless of the reason, are more likely to experience viral failure on therapy^56,57^ and less likely to achieve PTC if ART is stopped. Finally, frequency of monitoring HIV-RNA and assay variability are likely to affect blip detection, which may account for some of the observed differences in the significance and proportion of intermittent low-level viremia for ART-treated individuals.^8,9^ It is also possible that frequency of HIV-RNA monitoring could influence the definition of virologic failure rate in this analysis or clinical practise reflects concerns with ART adherence. The median number of HIV-RNA measurements per year on ART were similar to the frequency off ART [1 (1, 4.2) and 1.6 (1, 2.9), respectively]. We have attempted to correct for measurement frequency by including it as a variable in our Cox models. We were not, however, able to correct for assay variability because of the limited sample size and as it was unknown for >50% of HIV-RNA measurements. In addition, we did not distinguish between boosted and unboosted PIs but, to account for these unmeasured changes in treatment quality over time, we adjusted for ART initiation year. It is possible that newer more potent ART regimens, including integrase inhibitors, not routinely available at the time of this analysis, could additionally impact on size of reservoir and viral blips on therapy.^55^

Stopping ART within the setting of a cure study should be undertaken within close clinical and laboratory monitoring and extrapolation of observational data into a study design in terms of individual health risks and risks of onward viral transmission must be made with caution. Both individual potential risks and the risk of onward viral transmission, should viral rebound ensue, also need to be taken into account.

In conclusion, findings from this large observational cohort of treated seroconverters stopping ART indicate that the absence of viral blips >400 copies HIV-1 RNA/mL in individuals treated with early ART, close to the time of PHI diagnosis predicted a better chance of subsequent after treatment viremic control after ART cessation.

## APPENDIX 1 CASCADE Collaboration in EuroCoord

CASCADE Steering Committee: Julia Del Amo (Chair), Laurence Meyer (Vice Chair), H.C.B., Geneviève Chêne, Osamah Hamouda, Deenan Pillay, Maria Prins, Magda Rosinska, Caroline Sabin, Giota Touloumi.

CASCADE Co-ordinating Centre: K.P. (Project Leader), A.D.O., Andrea ARTier, Lorraine Fradette, Sarah Walker, Abdel Babiker.

CASCADE Clinical Advisory Board: H. C.B., Andrea De Luca, Martin Fisher, R.M.

CASCADE Collaborators: Australia PHAEDRA cohort (Tony Kelleher, David Cooper, Robert Finlayson, Mark Bloch) Sydney AIDS Prospective Study and Sydney Primary HIV Infection cohort (Tony Kelleher, Tim Ramacciotti, Linda Gelgor, David Cooper, Don Smith); Austria Austrian HIV Cohort Study (Robert Zangerle); Canada South Alberta clinic (John Gill); Estonia Tartu Ülikool (Irja Lutsar); France ANRS CO3 Aquitaine cohort (Linda Wittkop, Francois Dabis, Rodolphe Thiebaut), ANRS CO4 French Hospital Database (Dominique Costagliola, Marguerite Guiguet), Lyon Primary Infection cohort (Philippe Vanhems), French ANRS CO6 PRIMO cohort (Marie-Laure Chaix, Jade Ghosn), ANRS CO2 SEROCO cohort (Laurence Meyer, Faroudy Boufassa); Germany German HIV-1 seroconverter cohort (Osamah Hamouda, Claudia Kücherer, Barbara Bartmeyer); Greece AMACS (Anastasia Antoniadou, Georgios Chrysos, Georgios L. Daikos); Greek Haemophilia cohort (Giota Touloumi, Nikos Pantazis, Olga Katsarou); Italy Italian Seroconversion Study (Giovanni Rezza, Maria Dorrucci), ICONA cohort (Antonella d'Arminio Monforte, Andrea De Luca.) Netherlands Amsterdam Cohort Studies among homosexual men and drug users (Maria Prins, Ronald Geskus, Jannie van der Helm, Hanneke Schuitemaker); Norway Oslo and Ulleval Hospital cohorts (Mette Sannes, Oddbjorn Brubakk, Anne-Marte Bakken Kran); Poland National Institute of Hygiene (Magdalena Rosinska); Spain Badalona IDU hospital cohort (R.M., Jordi Tor), Barcelona IDU Cohort (Patricia Garcia de Olalla, Joan Cayla), CoRIS-scv (Julia del Amo, Santiago Moreno, Susana Monge); Madrid cohort (Julia Del Amo, Jorge del Romero), Valencia IDU cohort (Santiago Pérez-Hoyos); Sweden Swedish InfCare HIV Cohort, Sweden (Anders Sönnerborg); Switzerland Swiss HIV Cohort Study (H.C.B., Huldrych Günthard, Martin Rickenbach); Ukraine Perinatal Prevention of AIDS Initiative (Ruslan Malyuta); United Kingdom Public Health England (Gary Murphy), UK Register of HIV Seroconverters (K.P., Anne Johnson, A.P., Abdel Babiker), University College London (Deenan Pillay); African cohorts: Genital Shedding Study (US: C.M.; Family Health International, Robert Salata, Case Western Reserve University, Uganda: Roy Mugerwa, Makerere University, Zimbabwe: Tsungai Chipato, University of Zimbabwe); International AIDS Vaccine Initiative (IAVI) Early Infections Cohort (Kenya, Rwanda, South Africa, Uganda, Zambia: Pauli N. Amornkul, IAVI, USA; Jill Gilmour, IAVI, UK; Anatoli Kamali, Uganda Virus Research Institute/Medical Research Council Uganda; Etienne Karita, Projet San Francisco, Rwanda).

EuroCoord Executive Board: Fiona Burns, University College London, UK; Geneviève Chêne, University of Bordeaux, France; Dominique Costagliola (Scientific Coordinator), Institut National de la Santé et de la Recherche Médicale, France; Carlo Giaquinto, Fondazione PENTA, Italy; Jesper Grarup, Region Hovedstaden, Denmark; Ole Kirk, Region Hovedstaden, Denmark; Laurence Meyer, Institut National de la Santé et de la Recherche Médicale, France; Heather Bailey, University College London, UK; Alain Volny Anne, European AIDS Treatment Group, France; Alex Panteleev, St. Petersburg City AIDS Centre, Russian Federation; A.P., University College London, UK, K.P., University College London, UK; Claire Thorne, University College London, UK.

EuroCoord Council of Partners: Jean-Pierre Aboulker, Institut National de la Santé et de la Recherche Médicale, France; Jan Albert, Karolinska Institute, Sweden; Silvia Asandi, Romanian Angel Appeal Foundation, Romania; Geneviève Chêne, University of Bordeaux, France; Dominique Costagliola, INSERM, France; Antonella d'Arminio Monforte, ICoNA Foundation, Italy; Stéphane De Wit, St. Pierre University Hospital, Belgium; Peter Reiss, Stichting HIV Monitoring, Netherlands; Julia Del Amo, Instituto de Salud Carlos III, Spain; José Gatell, Fundació Privada Clínic per a la Recerca Bíomèdica, Spain; Carlo Giaquinto, Fondazione PENTA, Italy; Osamah Hamouda, Robert Koch Institut, Germany; Igor Karpov, University of Minsk, Belarus; Bruno Ledergerber, University of Zurich, Switzerland; Jens Lundgren, Region Hovedstaden, Denmark; Ruslan Malyuta (chair), Perinatal Prevention of AIDS Initiative, Ukraine; Claus Møller, Cadpeople A/S, Denmark; K.P., University College London, United Kingdom; Maria Prins, Academic Medical Centre, Netherlands; Aza Rakhmanova, St. Petersburg City AIDS Centre, Russian Federation; Jürgen Rockstroh, University of Bonn, Germany; Magda Rosinska, National Institute of Public Health, National Institute of Hygiene, Poland; Manjinder Sandhu, Genome Research Limited; Claire Thorne, University College London, UK; Giota Touloumi, National and Kapodistrian University of Athens, Greece; Alain Volny Anne, European AIDS Treatment Group, France.

EuroCoord External Advisory Board: David Cooper, University of New South Wales, Australia; Nikos Dedes, Positive Voice, Greece; Kevin Fenton, Public Health England, USA; David Pizzuti, Gilead Sciences, USA; Marco Vitoria, World Health Organisation, Switzerland.

EuroCoord Secretariat: Silvia Faggion, Fondazione PENTA, Italy; Lorraine Fradette, University College London, UK; Richard Frost, University College London, UK; Andrea ARTier, University College London, UK; Dorthe Raben, Region Hovedstaden, Denmark; Christine Schwimmer, University of Bordeaux, France; Martin Scott, UCL European Research & Innovation Office, UK.
